# Engineering Liquid-Vapor Phase Transition for Refreshable Haptic Interfaces

**DOI:** 10.34133/2022/9839815

**Published:** 2022-08-19

**Authors:** Wei Dawid Wang, Zhengbing Ding, Yongkyu Lee, Xu Han

**Affiliations:** Department of Mechanical Engineering, Hanyang University, Seoul 04763, Republic of Korea

## Abstract

Haptics as a communication medium has been increasingly emphasized across various disciplines. Recent efforts have focused on developing various haptic stimulation technologies; however, most of them suffer from critical drawbacks stemming from their bulk, complexity, large power input, or high cost. Here, we describe a strategy to design portable and affordable refreshable haptic interfaces composed of an array of individually addressable and controllable liquid pouch motor-based haptic units embedded in either rigid or flexible substrates for different application contexts. The pouch motor filled with low boiling fluid, under a controlled manner, expands or contracts by Joule heating or cooling, enabling the haptic pin in contact to be protruded or retracted. Programming the actuation sequence of an array of haptic units enables the haptic interface to apply different stimuli to the skin to convey corresponding information. We finally demonstrate the applications to portable rigid braille displays and flexible epidermal VR devices. This study opens the avenue to the design of ubiquitous refreshable haptic interfaces that is portable, affordable, scalable, and uninjurious.

## 1. Introduction

Haptic perception is one of the most important sensory abilities inherent to humans [1]. Humans can perceive various types of environmental stimuli through arrays of mechanoreceptors embedded at different depths of our skin [2–4], allowing us to achieve a high level of interaction with our surroundings. Due to the sensitivity of the skin, more and more attention is drawn to the development of haptic technologies for various applications [5–9] ranging from medical science to entertainment. There are especially two main underexploited social needs, i.e., portable and electronic braille displays [10–12] capable of providing diverse information for the visually impaired through a sense of touch and virtual reality (VR) [13–18] capable of recreating multiple artificial interactions to enable users to be fully immersed in an artificially constructed environment.

Recent advances in haptic interfaces are being developed based on various actuation techniques. Amid, vibrotactile, and electrotactile are the most commonly used forms of haptic actuation, but these techniques are difficult to use in braille applications and cannot provide the user with the type of displacement and persistence of touch for a natural experience [18–20]. On the other hand, soft robots based on soft and compliant materials, especially electrically driven soft actuators, have shown the greatest potential in the development of the haptic interface to meet its design requirements such as lightweight, compact design, low cost, large deformation, and distributed and localized actuation [21, 22]. However, various actuation methods among them, including piezoelectric actuators [23–25], electromagnetic actuators [26, 27], shape memory actuators [28, 29], electrostatic actuators [30], and dielectric elastomer actuators [31–33], suffer from critical drawbacks that stem from their bulk, complexity, large power input, or high cost. Hence, there is an urgent need to design a haptic interface that combines multiple advantages, including refreshable, portable, compact, painless, low-cost, easily scalable, and low power consumption.

**Figure 1:**
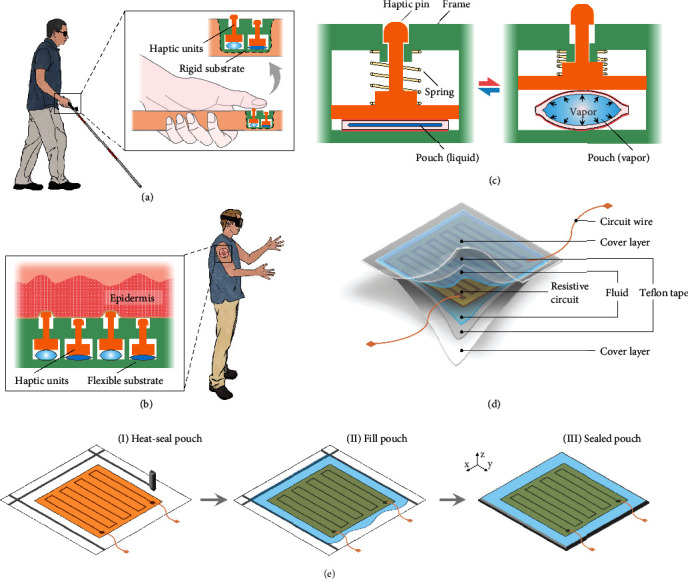
Potential applications and conceptual design of the refreshable haptic interfaces. (a) Example application of the braille display that provides diverse information for the visually impaired through a sense of touch. (b) Example application of flexible epidermal VR devices that provides different haptic stimulations. (c) The working principle of the refreshable haptic unit and its main components. Phase transition of the low boiling fluid inside the liquid pouch results in expansion and contraction of the liquid pouch, which protrudes and conceals the haptic pin. (d) Schematic diagram of liquid pouch motor indicating its constituent components. (e) The main fabrication process of the liquid pouch motor.

Here to satisfy these requirements, we introduce a refreshable haptic interface consisting of an array of liquid pouch motor-based millimeter-scale haptic units embedded in either rigid or flexible substrates with applications to rigid braille displays and flexible epidermal VR devices (Figures 1(a) and 1(b)). The haptic unit mainly consists of a haptic pin and a liquid pouch motor that is a gas-tight soft pouch filled with low boiling fluid and equipped with a built-in resistive circuit as he heating element. The liquid pouch motor expands or contracts, through the vapor-liquid phase transition of the filled fluid, by Joule heating the embedded resistance circuit or free cooling, enabling the haptic pin in contact to be protruded or retracted. Control strategies on the haptic unit were explored to improve the actuation frequency, prevent damage from prolonged heating, and accommodate a wide range of operating temperatures. Programming the actuation sequence of an array of haptic units enables the haptic interface to apply different stimuli to the skin to convey corresponding information. We finally extend our results to the design of pixelated haptic devices for applications of portable rigid braille cells that are attachable to common assistive devices for the visually impaired and flexible epidermal VR devices that can be attached to surfaces of the skin to produce multieffect interactions.

## 2. Working Principle and Conceptual Design

The proposed haptic interface is designed by embedding multiple haptic units into a rigid or flexible substrate according to different application contexts. The central component of the haptic interface is the haptic unit mainly consisting of a liquid pouch motor, a haptic pin, and a compression spring, where their relative positions are determined by the frame (Figure 1(c)). The pouch motor is an expandable gas-tight pouch, made of flexible film, filled with low boiling fluid, and equipped with a built-in resistance circuit as the heating element (Figure 1(d)). Joule heating of the resistive circuit to a temperature above the boiling point of the fluid enables its liquid to vapor phase transition. The heating process results in the inflation of the pouch, pushing the haptic pin upward to its protruded state that applies tactile stimuli to the user's skin. If the Joule heating is eliminated, free cooling enables the reverse vapor to liquid phase transition so that the inflated pouch contracts and the haptic pin retracts. The compression spring makes the haptic pin fit tightly with the pouch surface to avoid separation, which enables the deformation of the pouch motor to equal the displacement of the haptic pin. The protruded and retracted state of each unit's haptic pin can be independently controlled so that different display patterns, corresponding to different tactile modes, of the haptic interface composed of arrays of haptic units can be formulated by controlling desired haptic units.

The key component of the haptic unit is the liquid pouch motor. Its cover layer is made of the metallic film (aluminum vapor deposition film) without air permeability to avoid fluid loss over time, but many other heat-sealable materials and composites could also be used. The embedded resistive circuit is formed by copper traces on a polyimide film in filamentary serpentine patterns to maximize heat dissipation. A thin layer of Teflon tape is pasted to the inner side of the cover layer. This tape enables the heating element to avoid direct contact with the cover and prevent the cover from being damaged by overheating. The boiling point of the filled fluid is chosen as 61°C, which is higher than the temperature of the human living environment but will not harm the human body. Figure 1(e) shows the fabrication process of the liquid pouch motor following three main steps: sealing three sides of the pouch, filling engineered fluid and placing heating element, and completely sealing the pouch. The pouch motor is designed in a square shape with a side length of around 6 mm to enable the entire 6-dot braille cell can be covered by the finger pad (Figure S1). In addition, the braille display requires the stroke and generated force of the braille pin to be at least 0.6 mm and 50 mN to avoid any misreading [34]. On the other hand, the liquid pouch motor for the VR devices continues the design parameters of that for the braille cell, for the stroke and generated force of the braille pin enables the skin to have a distinct sense of touch without causing pain to the skin.

## 3. Performance Characterization of Liquid Pouch Motor

**Figure 2:**
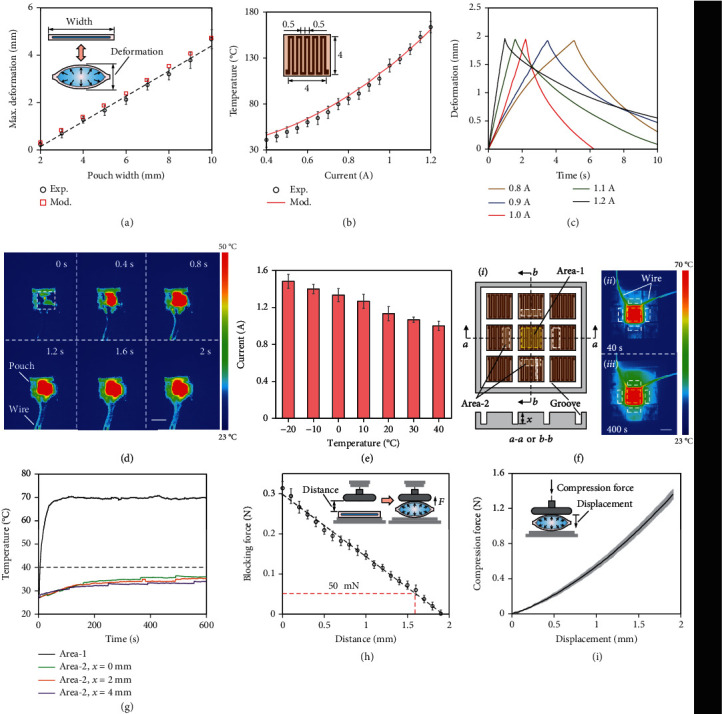
Performance evaluation of the liquid pouch motor. (a) Experimental and modeling results of the maximum inflated deformation of the square pouch with different side lengths. Inset shows the schematic of the liquid pouch motor before and after inflation. (b) The experimental and modeling results of the temperature of the heating element after applying different electric currents at 2 s. Inset shows the schematic of the heating element and its main dimensions in millimeters. The copper trace etched onto a polyimide sheet is 0.5 mm wide and 18 *μ*m thick and consists of a serpentine pattern to maximize heat dissipation. (c) The time-displacement curve of the liquid pouch motor for different values of applied current. (d) Thermal infrared images depicting the thermal change in the pouch motor during its inflation within 2 s. (e) The required actuation current that satisfies predefined displacement conditions under different ambient temperatures. (f) Thermal interference test. The array of heating elements (*i*) and its thermal infrared images at 40 s (*ii*) and 400 s (*iii*) when area 1 is maintained at 70°C. One yellow dotted box represents area 1 and four white dotted boxes represent area 2s. (g) Average temperatures of area 1 and area 2 over time. (h) The blocking force of the liquid pouch at different initial offset distances at 2 s when a current of 1 A was applied. Insets show the schematic of the experimental setup. (i) The liquid pouch's resistivity to compression. Inset shows the schematic of the experimental setup. All scale bars: 5 mm.

Experiments were first conducted to evaluate the effect of the side length of the squared pouch on its maximum inflated deformation. The side length of the pouch varied from 2 mm to 10 mm in increments of 1 mm. The pouches were then immersed in hot water of 65°C to guarantee instant and maximum inflation of the pouches. The results show that the maximum deformation of the pouch changes approximately linearly with the side length (Figure 2(a), Figure S2). In reality, the heating element, with an electrical resistance of 0.65 *Ω*, was embedded in the pouch to trigger the liquid to vapor phase change of the filled fluid by Joule heating. Figure 2(b) shows the temperatures of the heating element at 2 s after different electric currents were applied, under an ambient temperature of 40°C (Figure S3). The current determines the temperature of the heating element and therefore the speed at which the pouch motor expands. Figure 2(c) illustrates the deformation curves of the pouch motor with a side length of 6 mm, under 40°C, for different currents until its maximum deformation of around 1.93 mm that can fully provide the required displacement by the mentioned haptic pin. The pouch is filled with around 1 *μ*L of fluid to ensure that the pouch can be fully inflated, and it can also ensure that the internal pressure after inflation will not damage the pouch (Figure S4). One can see that a larger current causes rapid deformation of the pouch, while it also generates excessive heat and prolongs the cooling time. Conversely, applying a lower current requires a longer heating time, which also leads to excessive heat according to Joule's first law. Consequently, the current value of 1.0 A, i.e., an electrical power of 0.65 W, was determined to actuate the liquid pouch motor, and it can fully inflate the pouch motor in a relatively short time of about 2 s. Figure 2(d) shows thermal infrared images depicting the thermal change in the pouch motor during its inflation through Joule heating of the internal heating element.

The ambient temperature also affects the deformation performance of the pouch motor. We have tested the performance of the same liquid pouch motor ranging from -20°C to 40°C, in which the temperature of the most human living environment falls within. Figure 2(e) shows the required current that satisfies the conditions that to reach a maximum deformation of 1.93 mm under 2 s (see Figure S5). One can see that a lower ambient temperature requires a higher current to compensate for faster heat dissipation. However, in connection with the showings of Figure 2(b), we note that at certain ambient temperatures, the use of extreme current might lead to pouch failure and should be avoided.

The pouch motors are inflated by Joule heating, and the thermal interference between adjacent units should be investigated. Nine square heating elements of 6 mm side length (i.e., the size of pouch motor) are arranged in a three-by-three array, with two adjacent heating elements separated by grooves having the same width of 1 mm but different depths of 0, 2, and 4 mm, respectively (Figure 2(f)). Consider that the temperature to inflate the pouch motor is 70°C, so the temperature of the central heating element (i.e., area 1 in Figure 2(f)) is kept at 70°C, and its thermal effect on the adjacent heating elements is observed by using a thermal camera (Figure S6). Figure 2(f) also describes the thermal infrared images at 40 s and 400 s. One can see the side of the adjacent elements close to the central element will have a higher temperature than the other side. Therefore, the average temperature of the high-temperature area, i.e., the one-third area of adjacent elements closing to the center element indicated as area 2, is measured. Figure 2(g) describes the measured average temperatures of area 1 and area 2s, and results show that the temperature of 70°C in the central element caused the temperature of the adjacent elements to increase and eventually maintain at a temperature of around 35.5°C, 33.9°C, and 33.2°C for different depths of the groove of 0, 2, and 4 mm, respectively. The results also show that the grooves can further reduce the thermal interference between adjacent units where a deeper depth of the groove results in a lower maintained temperature.

Force characteristics of the same liquid pouch motor were also examined (see Supplementary Materials for experimental setups). The first experiment was conducted to measure the blocking force on a fixed surface, comparable to the scenario of the skin waiting for the display pins to be felt. Figure 2(h) shows the recorded maximum force, and the results show that as the amount of deformation increases, the blocking force continues to decrease, and 50 mN is obtained at the expansion deformation of around 1.6 mm. The second experiment was conducted to measure the compression force that the fully inflated pouch can withstand, comparable to the scenario of protruded pins that withstand external force. The fully inflated pouch is compressed, and results show that the compression force continues to increase as the compressed distance increases, and the compression force is significantly greater than the blocking force (Figure 2(i)).

## 4. Control Strategy of Single Haptic Unit

**Figure 3:**
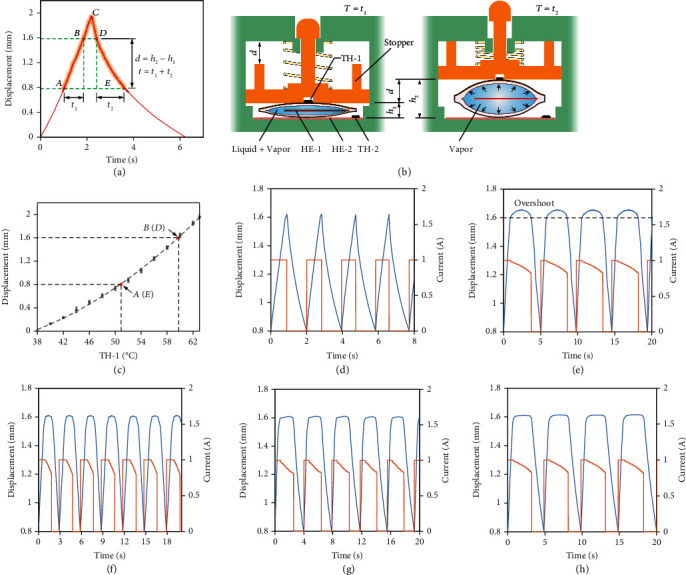
Control strategy of the single haptic unit. (a) The time-displacement curve of the liquid pouch motor with a side length of 6 mm under the applied current of 1 A at an ambient temperature of 40°C. The highlighted portion indicates the region of interest between the displacements of *h*_1_ = 0.8 mm and *h*_2_ = 1.6 mm in approximately *t* = *t*_1_ + *t*_2_ = 2 s. This region was intentionally selected to enable faster repetitive actuation while still satisfying the minimum required force and displacement. (b) The schematics of the single haptic unit before and after actuation. (c) The corresponding relationship between the amount of displacement of the haptic pin and the temperature detected by TH-1 when the base heating element is maintained at 40°C. The inflated deformation of the pouch occurs from about 38°C. (d) The applied current pattern (in red) and the corresponding reciprocating motion (in blue) of the haptic pin with the stroke of 0.8 mm at around 0.5 Hz. (e) The applied current pattern and the corresponding reciprocating motion of the haptic pin with certain overshoot while maintaining peak position for 3 s in each cycle. (f–h) The applied current pattern (in red) and the corresponding reciprocating motion (in blue) of the haptic pin without overshoot while maintaining peak position for 1 s, 2 s, and 3 s in each cycle, respectively.

Despite promising qualities, the low actuation frequency within 0.1 Hz is one of the most critical drawbacks of liquid pouch motors that depend on phase transition for actuation [35, 36]. A control strategy is introduced here to enhance the performance of a single haptic unit. Since the slow cooling process of a liquid pouch motor around the initial inflated deformation accounts for the majority of the time consumed in a single actuation cycle (Figure 3(a)), minimizing the cooling process is the key to improve actuation frequency. This is achieved by reheating the pouch at a required displacement *d* labeled in Figure 3(a), defined as the difference between the two pin positions before it returns to its initial displacement. That is, by repeatedly actuating the highlighted portion in Figure 3(a), the actuation frequency can be improved several times, or even by an order of magnitude, under the premise of sacrificing an unnecessary part of the total deformation.

The deformation of the pouch motor was affected by both the temperature of the heating element (HE-1) embedded in the pouch (i.e., the magnitude of the applied current) and the ambient temperature. To implement the mentioned control strategy, a base heating element (HE-2) and two thermistors (TH-1 and TH-2) are added to the haptic unit to maintain the pouch motor at any desired deformation (Figure 3(b)). To eliminate the influence of the ambient temperature, HE-2 is placed outside of the liquid pouch motor to maintain the base plate at 40°C with the TH-2 as the feedback sensor to create similar conditions throughout, regardless of the ambient temperatures. This method guarantees the single cycles of the pouch, under different ambient temperatures ranging from -20°C to 40°C, can have similar performances (Figure S5). Then, the inflated deformation of the pouch motor can be determined by only controlling the temperature of the pouch motor detected by the TH-1 as the feedback to the HE-1 to adjust the applied current (Figure S7). Figure 3(c) shows the corresponding relationship between the inflated deformation amount of the pouch motor (i.e., the displacement of the haptic pin) and the temperature detected by TH-1 that are one-to-one positively related. That is, by setting the desired temperature value to TH-1, any corresponding position of the haptic pin can be achieved. By trial and error, the approximate temperatures at 0.8 mm and 1.6 mm from the initial position were approximately 51.5°C and 60°C, respectively.

The control strategy enables the haptic pin to achieve faster repetitive movement with the desired displacement interval (i.e., the stroke). For a specified stroke, when the temperature at the lower position is detected, a current (of 1 A) will flow through HE-1 to heat and inflate the pouch. When the temperature at the higher position is detected, the current will be linearly reduced to maintain the displacement in the desired time interval. Then, it can be cut off to make the inflated pouch contract through free cooling until it drops to the lower position, followed by the beginning of the next cycle. Figure S8 describes the haptic pin actuated at a frequency of 1 Hz with a stroke of 0.43 mm. The highlighted portion AB and DE of the curve in Figure 3(a) (satisfying the minimum required force of 50 mN) is selected for the proposed applications, which enables the haptic unit to be actuated with a stroke of 0.8 mm at the frequency of 0.5 Hz (Figure 3(d)). Figure 3(e) shows the haptic pin maintained at the higher displacement of 1.6 mm with a certain overshoot for a time interval of 3 s for each cycle. To reduce the overshoot, a limiting stopper is added to the haptic pin to ensure its accurate displacement *d* (Figure 3(b)). Figures 3(f)–3(h) show the improved repeated actuation of the haptic pin with a stroke of 0.8 mm and a dwell time of 1 s, 2 s, and 3 s at its higher position, respectively. The persistence of the actuation of the haptic unit was tested by continuous actuation for 1800 s, and the performance of the haptic unit has no observed degradation. The first 200 s of repetitive actuation of the haptic pin with different dwell times are depicted in Figure S9.

## 5. Refreshable Braille Display

**Figure 4:**
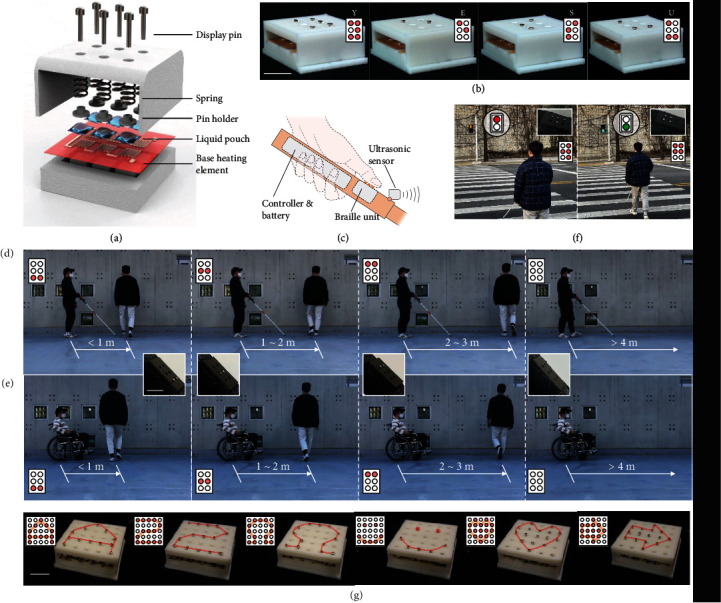
Application to the refreshable braille display. (a) Exploded-view schematic illustration of a six-dot braille haptic display consisting of six independently controlled haptic units. (b) Demonstration of different braille alphabets displayed on the braille display. Insets show both the corresponding schematic and Roman letters of the displayed patterns. (c) Schematic of the portable braille handle composed of the braille display, an ultrasonic sensor, a built-in control circuit, and batteries. Demonstration of the portable braille handle when attached to (d) a walking stick and (e) a wheelchair. Four different patterns can be displayed depending on the distance to the closest obstacle ahead. Insets show both the corresponding schematic and actual diagrams of the displayed patterns. (f) Potential application of the portable braille handle to provide the visually impaired with the haptic patterns according to different traffic light information for safely crossing the crosswalk. Insets show both the corresponding schematic and actual diagrams of the displayed patterns. (g) The refreshable braille cell can easily be scaled-up to display a variety of symbols and characters. Insets show the corresponding schematic of the displayed patterns. All scale bars: 10 mm.

As a demonstration for braille application, a six-dot braille haptic display consisting of six haptic units is designed, and Figure 4(a) shows the components of the haptic display. A monolithic heating element is located below the liquid pouch motors to maintain the base at 40°C. The six liquid pouch motors can be independently controlled, to drive their corresponding pins to move up and down, thereby being capable of achieving 64 (i.e., 2^6^) displayable patterns of the braille display. The distance between braille dots is determined as 7 mm, which enables the six dots to accommodate the size of an adult's fingertip. Figure 4(b) shows the demonstration of the braille display displaying different braille alphabets (Figure S10). The braille display is then introduced to the design of a portable braille handle, with a built-in control circuit and battery, which can be directly attached to common assistive devices such as the walking cane and the wheelchair. The braille handle can be used to detect the distance of obstacles by using an ultrasonic sensor and then transmit the detected obstacle information to the user through the braille haptic display (Figure 4(c)). Four patterns of the braille haptic display are designed to be displayed based on the detected different distances, with application to a walking cane and a wheelchair separately (Figures 4(d) and 4(e), Movie S1). The functionality of the braille handle can be expanded (beyond merely distance sensing devices), and Figure 4(f) shows the potential application of the portable braille handle to provide the visually impaired with the haptic patterns according to different traffic light information for safely using the crosswalk, once the traffic light information can be accessed through modern GPS technologies. Next, we demonstrate the scalability by introducing a 5 by 5 scaled-up haptic display. Scaling up is relatively straightforward due to the modular design, and more elaborate patterns are shown through the scaled-up display in Figure 4(g).

## 6. Flexible Epidermal VR Device

**Figure 5:**
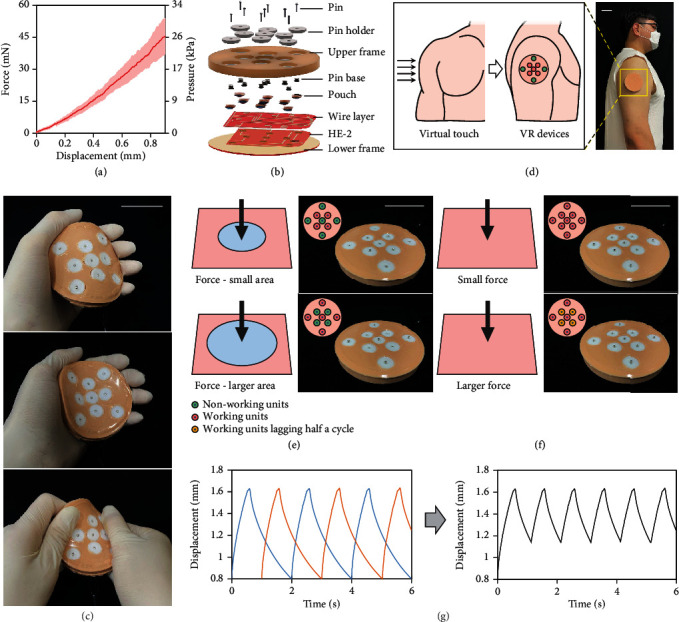
Application to flexible epidermal VR devices capable of providing different haptic stimulations. (a) Deformation-resisted force response of a pin in contact with the arm skin. (b) Exploded-view schematic illustration of the VR device composed of nine independently controlled haptic units. (c) Optical images of the VR device under bending, folding, and twisting. (d) The flexible VR device can be used to generate virtual touch on human skin. (e) The VR device can adjust the target area to simulate forces with different applied areas. (f) The VR device can provide different frequencies of stimulation to simulate applied force with different magnitudes. Units in red are actuated, units in green are in rest, and units in orange indicate units that are actuated half a cycle apart from the red units. (g) The use of intentional time lag of certain pins creates the effect of improved actuation frequency for indicating external forces with a larger magnitude. All scale bars: 30 mm.

In contrast to the haptic units enclosed in rigid frames, we further explore the capabilities of the haptic units by embedding them in a flexible matrix to create an epidermal VR device that provides haptic stimulations in the form of various virtual touch.  Figure 5(a) describes the force response of the human skin of the upper arm, before the obvious pain occurs, by displacement in contact with the pin used for the haptic unit, which is still well within the range of forces that can be generated through the liquid pouch motor described in Figure 2(h).  Figure 5(b) presents schematic illustrations of the epidermal VR device, and its fabrication process is shown in Figure S11. The device contains a base heating element to heat the base to the desired temperature regardless of ambient temperatures. In addition, a wire layer is included to facilitate the connection between the heating elements inside the pouch and the external control circuit. The silicone upper and lower silicone frames enable to create a flexible interface with the desired amount of stiffness. Due to the structural flexibility of the whole device, the device can bend, fold, and twist freely according to the target surface allowing it to be easily attached to the skin with thin silicone tape (Figures 5(c) and 5(d)).

The epidermal VR device, consisting of nine independently controlled haptic units, is capable of producing different display patterns to mimic different tactile effects (Figure S12). Our skin enables people to feel deformations of different types and intensities. To give different irritation to the skin, four different display patterns are then described to simulate four force conditions, namely, large-area force, small-area force, small force, and large force (Figures 5(e) and 5(f)). These patterns can be realized by dividing the nine independent units into two groups. The center unit and the four peripheral units form the first group, and the other four units form the second group. The virtual touch that realizes large-area force and small-area force is realized by actuating the first group of haptic units and the second group of haptic units, respectively. That is because the area enclosed by the units of the first group is larger than that enclosed by the units of the second group. Besides, the virtual touch that realizes a small force and a large force is realized by actuating the two groups of units synchronously and alternately with a half-cycle difference, respectively (Movie S2). That is, the large force is achieved by doubling the actuation frequency, on the premise of sacrificing partial displacement of the haptic pin (Figure 5(g)).

## 7. Conclusion

In summary, we introduced the liquid pouch motor based on liquid-vapor phase transition to the design of a refreshable haptic interface with applications to braille displays and epidermal VR devices that are representatives of a broad spectrum of potential applications. The actuation force and frequency of the pouch motor can be customized by determining the size of the pouch and purposefully utilizing different deformation stages of the pouch motor. By selecting the rapid deformation stage in a cycle, the available actuation frequency of the pouch motor has been increased several times, or even increased by an order of magnitude, compared with previous studies, enabling the refresh frequency of the haptic interface is within an acceptable range. The small size liquid pouch motor can be used to design the millimeter-scale haptic unit, and arrays of independently controlled millimeter-scale haptic units further have the potential to design small-resolution pixelated haptic interfaces. Due to the modular design, the haptic interfaces can be easily scalable by integrating many haptic units into either rigid or flexible substrates to meet the needs of various applications. Take the flexible epidermal device as an example, in principle, the modular design enables the size of flexible haptic interfaces to be extended to length scales or large areas that match the human body. These haptic interfaces can be directly attached to the skins of different parts of the body, such as the chest, back, arms, and legs, to generate haptic stimulations in the form of various virtual touches. Moreover, the haptic interface is electronically actuated by low voltage; thus, it can be applied to portable devices without the need for cumbersome appendages. Besides, the easy availability of materials and ease of fabrication also enable low production cost at different scales. Lastly, the liquid pouch motor as one of the soft robotic actuators enables the haptic interface with inherent advantages of the soft robot, capable of providing soft and harmless tactile sense. Altogether, our study opens pathways for the creation of ubiquitous haptic interfaces that are compact, portable, affordable, scalable, and uninjurious.

## Data Availability

All data needed to evaluate the conclusions in the paper are presented in the paper and/or the Supplementary Materials. Additional data related to this paper may be requested from the authors.

## Supplementary Materials

Supplementary Materials Supplementary 1. Note S1: materials and methods. Figure S1: determination of the size of the liquid pouch motor. Figure S2: experimental and modeling results of the maximum inflated deformation of the square pouch. Figure S3: modeling results of the maximum temperature of the heating element under different currents. Figure S4: internal pressure test of pouch motor. Figure S5: deformation of liquid pouch motor under the same base temperature. Figure S6: experimental setup for thermal interference test. Figure S7: circuit diagrams for braille haptic devices. Figure S8: haptic unit with an actuating frequency of 1 Hz. Figure S9: reciprocating motion of the haptic pin. Figure S10: different display characters corresponding to Roman letters. Figure S11: fabrication process of the flexible epidermal VR device. Figure S12: control diagrams for the epidermal VR device. Table S1: main properties of the engineered fluid. Supplementary 2. Movie S1: haptic patterns of the braille handle. Supplementary 3. Movie S2: haptic patterns of the flexible epidermal VR device.
