# Supplementary material for: Engineering Liquid-Vapor Phase Transition for Refreshable Haptic Interfaces
Source: Research (Wash D C). 2022 Aug 19;2022:9839815. doi: 10.34133/2022/9839815 (PMC9429979; doi:10.34133/2022/9839815)
Supplement: Supplementary Materials — Supplementary 1. Note S1: materials and methods. Figure S1: determination of the size of the liquid pouch motor. Figure S2: experimental and modeling results of the maximum inflated deformation of the square pouch. Figure S3: modeling results of the maximum temperature of the heating element under different currents. Figure S4: internal pressure test of pouch motor. Figure S5: deformation of liquid pouch motor under the same base temperature. Figure S6: experimental setup for thermal interference test. Figure S7: circuit diagrams for braille haptic devices. Figure S8: haptic unit with an actuating frequency of 1 Hz. Figure S9: reciprocating motion of the haptic pin. Figure S10: different display characters corresponding to Roman letters. Figure S11: fabrication process of the flexible epidermal VR device. Figure S12: control diagrams for the epidermal VR device. Table S1: main properties of the engineered fluid. Supplementary 2. Movie S1: haptic patterns of the braille handle. Supplementary 3. Movie S2: haptic patterns of the flexible epidermal VR device. [file 9839815.f1.zip › Supplementary Material.docx]

**Supplementary Materials**

**Engineering liquid-vapor phase transition for portable and electronic haptic interface**

Wei Dawid Wang, Zhengbing Ding, Yongkyu Lee, and Xu Han

Department of Mechanical Engineering, Hanyang University, Seoul 04763, South Korea

Correspondence should be addressed to Wei D. Wang; [davidwang@hanyang.ac.kr](mailto:davidwang@hanyang.ac.kr)

**Note S1. Materials and Methods**

**Materials and fabrication.** The heat-sealable metallic film (aluminum vapor deposition film) with a thickness of 0.075 mm is prepared as the cover layer of the pouch motor. The metal film is composed of three layers of thin-film materials. They are polyethylene terephthalate (PET), an aluminum film, and cast unoriented polypropylene (CPP). The heat resistance of the metal material composed of these three layers is about 135 °C. All the heating elements, fabricated via etching, are formed by copper traces (with a thickness of 18 $\mu$m) on a polyimide (with a thickness of 13 $\mu$m) in filamentary serpentine patterns to maximize heat dissipation. Figure 1e shows the fabrication process of the liquid pouch motor following three basic steps: sealing three sides of the pouch and pasting a thin Teflon tape to the inner side of the pouch cover layer, filling engineered fluid and placing heating element, and completely sealing the pouch. The main characteristics of the used engineering fluid are shown in Table S1. For the refreshable braille display, the rigid frame structure is made of polylactic acid (PLA) fabricated through a fused deposition modeling (FDM) 3D printer (Guider II, Flashforge 3D Technology). For the flexible epidermal VR device, both the upper and bottom frames are fabricated using Ecoflex 00-50 (Smooth-On).

**Modeling and simulation.** Finite element simulations were conducted to predict the maximum inflated deformation of the liquid pouch motor by using the commercially available finite element software package ABAQUS (Dassault Systèmes). The internal surfaces of the fully actuated pouch motor were assumed to be subjected to uniform pressure, and the pouch shell was meshed using linear quadrilateral elements. The geometrical configuration and boundary conditions of the finite element models were the same as those of the samples used in the experiment.

**Force experiments of liquid pouch motor.** The blocking forces of the liquid pouch motor were measured by maintaining the distance between the pouch and the load cell between 0 mm to 1.6 mm and adjusting the height of the load cell in increments of 0.1 mm. The pouch is then fully actuated under an applied current of 1 A for 2 s, and the maximum force is recorded. All the experiments were conducted by using a universal testing machine (AGS-X Series, Shimadzu) with a 5-N load cell (SMT1-5N, Interface) connected to a plate. Using the same machine, the compression forces were measured and recorded by using the same plate with a decreasing speed of 150 mm/min to compress the fully inflated pouch motor.

**Mathematical model of liquid pouch motor.** The volume of the liquid injected into the pouch is significant for Liquid Pouch Motors. The pouch cannot fully inflate into the theoretical maximum volume if it is too small, while the actuator's weight and cost increase as they were injected too much. Here, We developed a mathematical model of the pouch motor Fig.S2b. The initial state of the pouch with no pressure is a flat square, and the geometry of the pouch under positive pressure is an airfoil shape. We derive the minimum volume of liquid substance $V_{a}$ needed for the full actuation.

Given the maximum volume of the pouch filled with gas $V_{g}$, the maximum amount of gas substance $n_{g}$ [mol] in a pouch is calculated as follows, using the ideal gas state equation $PV=nRT$,

$n_{g}$= $\frac{P}{RT}V_{g}$ (1)

Letting *M* [g/mol] be the molar mass of the substance, and the mass of the substance *m* [kg] is derived as

*m*=${Mn}_{g}\times{10}^{-3}$ (2)

And let the density of the liquid substance be $\rho_{g}$[kg/m^3^], so $V_{a}$ is

$V_{a}$= $\frac{m}{\rho}$

= $\frac{{Mn}_{g}\times{10}^{-3}}{\rho}$

=$\frac{M}{\rho}\frac{P}{RT}V_{g}$* (3)

If pressured membranes try to maximize the cross-sectional area of the pouch. Assuming the surfaces of the pouch are cylindrical, we obtain equations eq.(4), eq.(5), and eq.(6)

$L_{\theta}$= 2*rθ* (4)

*r*$\sin\theta$=$\frac{L}{2}$ (5)

*S* = 2$r^{2}\theta$-*Lr*$\cos\theta$ (6)

where $L_{\theta}$ is an initial length when the pouch is flat, r is the radius of the curve of the membrane, θ is the central angle of the circular segment, L is the length of the chord or the length of the pouch, and S is the cross-sectional area of the pouch. From eq.(4) and eq.(5), by eliminating the radius r, we obtain the length of the pouch, that is

$L_{\theta}$=$L_{0}\frac{\sin\theta}{\theta}$ (7)

The volume of the pouch $V_{a}$ can be derived as

$V_{a}$=$\frac{L_{0}^{2}D}{2}$($\frac{\theta-\cos\theta\sin\theta}{\theta^{2}}$) (8)

where *D* is the width of the pouch. We have obtained both the length and the volume of the pouch as a function of the parameter *θ*, and *L*_0_ and *D* are constant. Especially, when the shape of the pouch is rectangular, *θ* = $\frac{\pi}{2}$ Eq. (3) is expressed as follows using Eq. (8).

$V_{a}$= $\frac{M}{\rho}\frac{P}{RT}V(\frac{\pi}{2})$ ×${10}^{-3}$ (9)

=$\frac{M}{\rho}\frac{P}{RT}\frac{L_{0}^{2}}{\pi}$ ×${10}^{-3}$ (10)

For example, when we substitute *M* = 250, *ρ* = 1510 (corresponding to Novec 7100) and $L_{0}$= 0.006 m, *D* = 0.006 m into Eq. (10), the minimum volume of liquid needed is calculated as around 0.4 *μ*L under the condition that the temperature and the pressure are normal (*T* = 273.15, *P* = 101325, and *RT*/*P* = 22.4×${10}^{-3}$). *θ* reaches the maximum (*θ* = $\frac{\pi}{2}$).


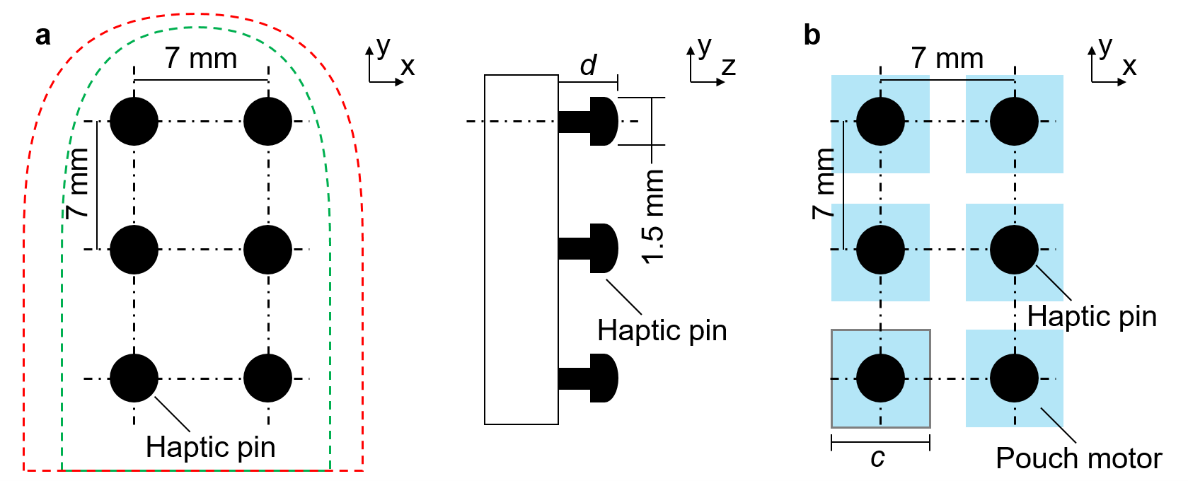


**Figure S1. Determination of the size of the liquid pouch motor.** Schematic of a six-dot braille haptic display consisting of six haptic units. The entire 6-dot braille cell should be covered by the finger pad, which leads to the maximum distance between two adjacent haptic pins to be approximately 7 mm. The dash lines in red and green indicate the outlines of the distal segment of the thumb and the index finger, respectively. In addition, braille displays require the minimum displacement of the haptic pins to be approximately 0.6 mm. However, by taking into account a safety factor when calculating the required displacement, the displacement is determined as *d* = 0.8 mm in this study. The distance between two adjacent haptic pins results in the maximum side length *c* of the pouch should not exceed 7 mm. Taking into account the need for the gap between adjacent pouches, the maximum side length is determined to be *c* = 6 mm.

**a**


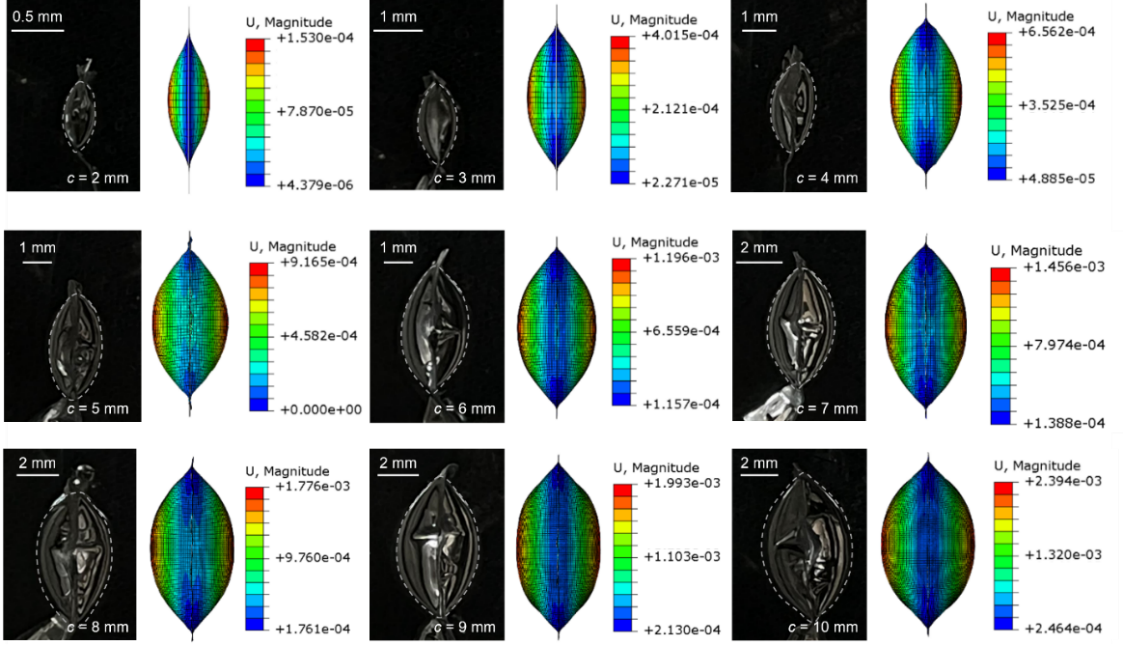


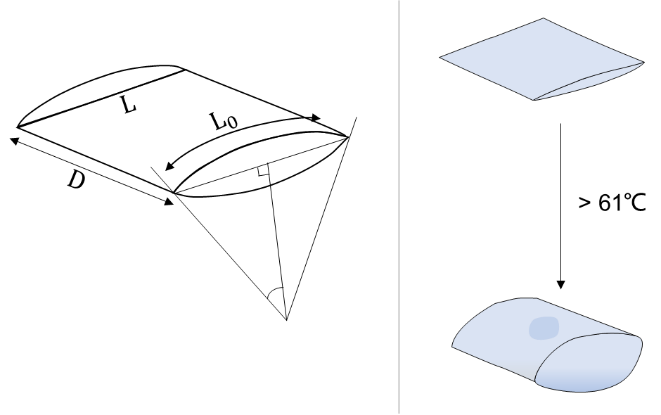
**b**

**Figure S2. Experimental and modeling results of the maximum inflated deformation of the square pouch with different side lengths.** **a**, The side length c ranges from 2 mm to 10 mm, in 1 mm increments. The photos of the fully inflated pouch motor were taken to measure the deformation of the pouch motor using an image analysis program (ImageJ, version 1.50d). **b**, Geometric parameters for a single pouch.


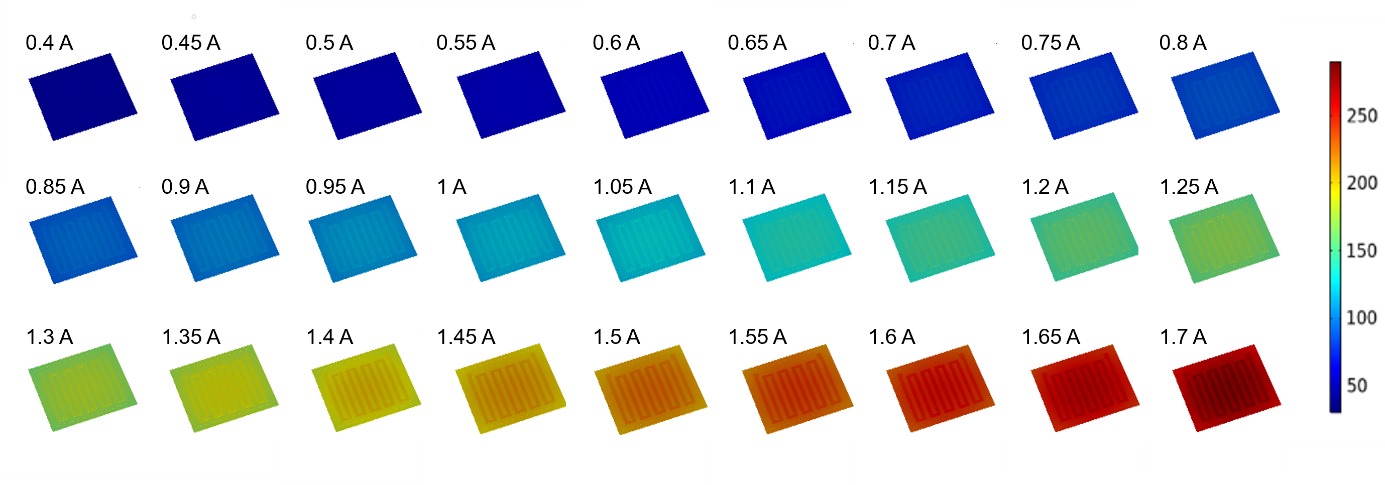


**Figure S3. Modeling results of the maximum temperature of the heating element under different currents**. Results show the modeling temperature (COMSOL Multiphysics 5.6) of the heating element after applying different electric currents at 2 s.


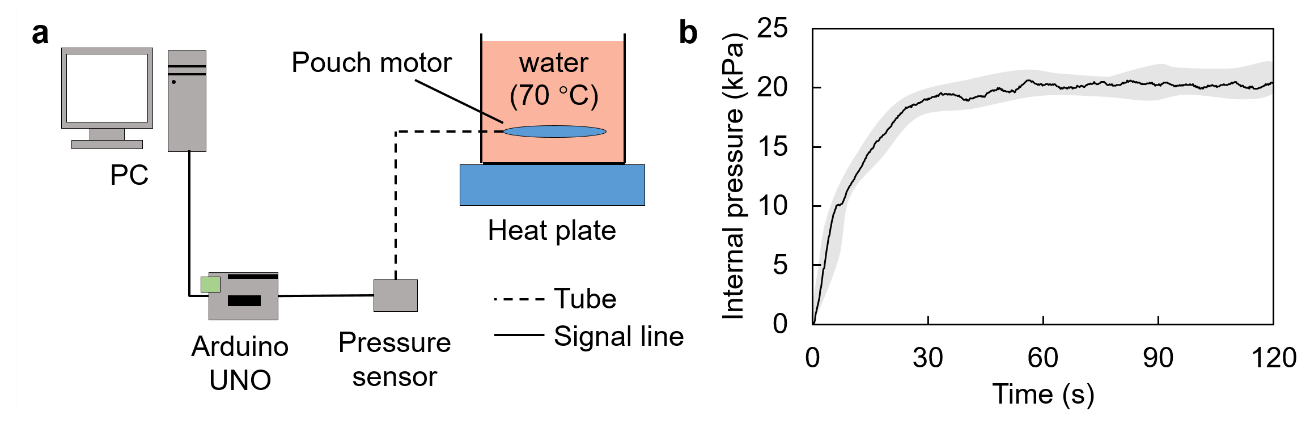


**Figure S4. Internal pressure test of pouch motor. a,** Circuit diagram of the experiment setup. The pouch motor filled with 10 $\mu$L of the fluid is connected to a pressure sensor (33A-015G-2210, Shiba Korea) via a thin and flexible tube. The pouch is then immersed in water at 70 ℃, and the change of the internal pressure of the pouch motor is read through a microcontroller (Arduino UNO). **b**, The time-internal pressure curve of the pouch motor, with a maintained maximum pressure of about 20 kPa. The entire pouch was immersed in water at 70° for more than an hour without leakage or explosion. It is noted that the pouch motor used here is filled with 10 $\mu$L of fluid instead of 1 $\mu$L, that is because there will always be volatilization of the solution during the preparation of the experimental setup, which has a great impact on the solution of 1 $\mu$l. In addition, the current pouch motor is designed to be capable of withstanding the pressure created by 10 $\mu$L of the filled fluid without leakage or explosion, and therefore the pressure created by the 1 $\mu$L of the fluid.


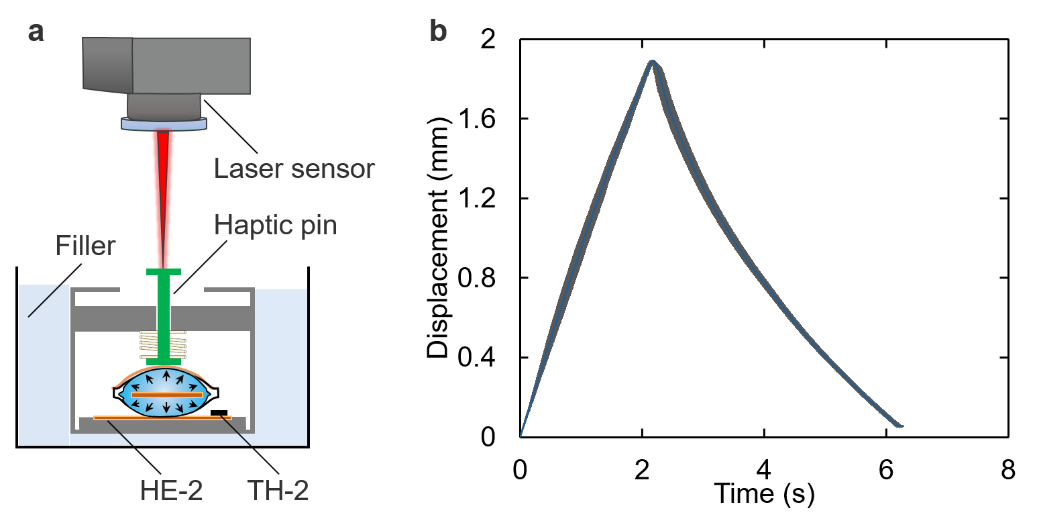


**Figure S5. Deformation of liquid pouch motor under the same base temperature. a,** Schematic of the experiment setup. The displacement of the haptic pin is measured and recorded by using a laser sensor (RF603, Riftek). The filler is used to adjust the ambient temperature ranging from -20 ℃ to 40 ℃, and the filler can be a mixture of ice and salt, a mixture of ice and water, or warm water. Different sub-zero temperatures are obtained by using the mixture of ice and salt through different mass ratios of ice and salt. This method allows the obtained lowest temperature to be lower than -20°C. The ambient temperatures of 0 ℃ and 10 ℃ are obtained using the mixture of ice and water. The ambient temperature above 10 ℃ is adjusted with warm water. **b**, The time-displacement curve of the haptic pin, maintaining the temperature of the base heating element (HE-1) at 40 ℃, under various ambient temperatures varied from -20 ℃ to 40 ℃ in increments of 10 ℃.


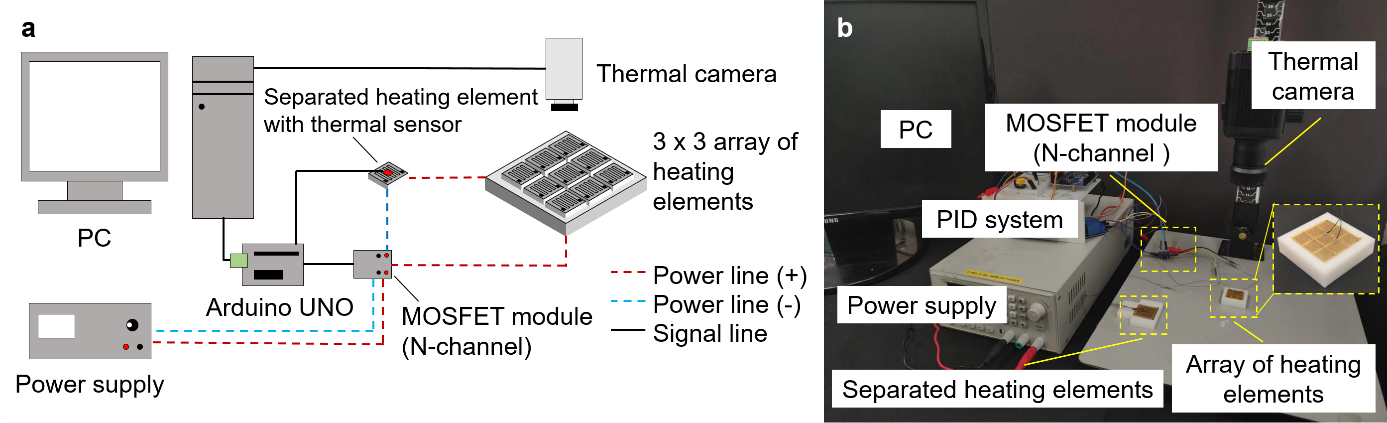


**Figure S6. Experimental setup for thermal interference test. a,** Circuit diagram of the experimental setup. **b,** Physical experimental setup. A separated heating element installed with a thermal sensor (NTC-10K) is connected in series to the centered heating element of the three-by-three array of heating elements. The two heating elements connected in series will have the same temperature via Joule heating. A PID control system based on a microcontroller board of Arduino UNO and a MOSFET module is applied to maintain the temperature of the centered heating element at 70 ℃. A thermal camera (CG640, Cox) is used to detect the temperature change of the array of heating elements.


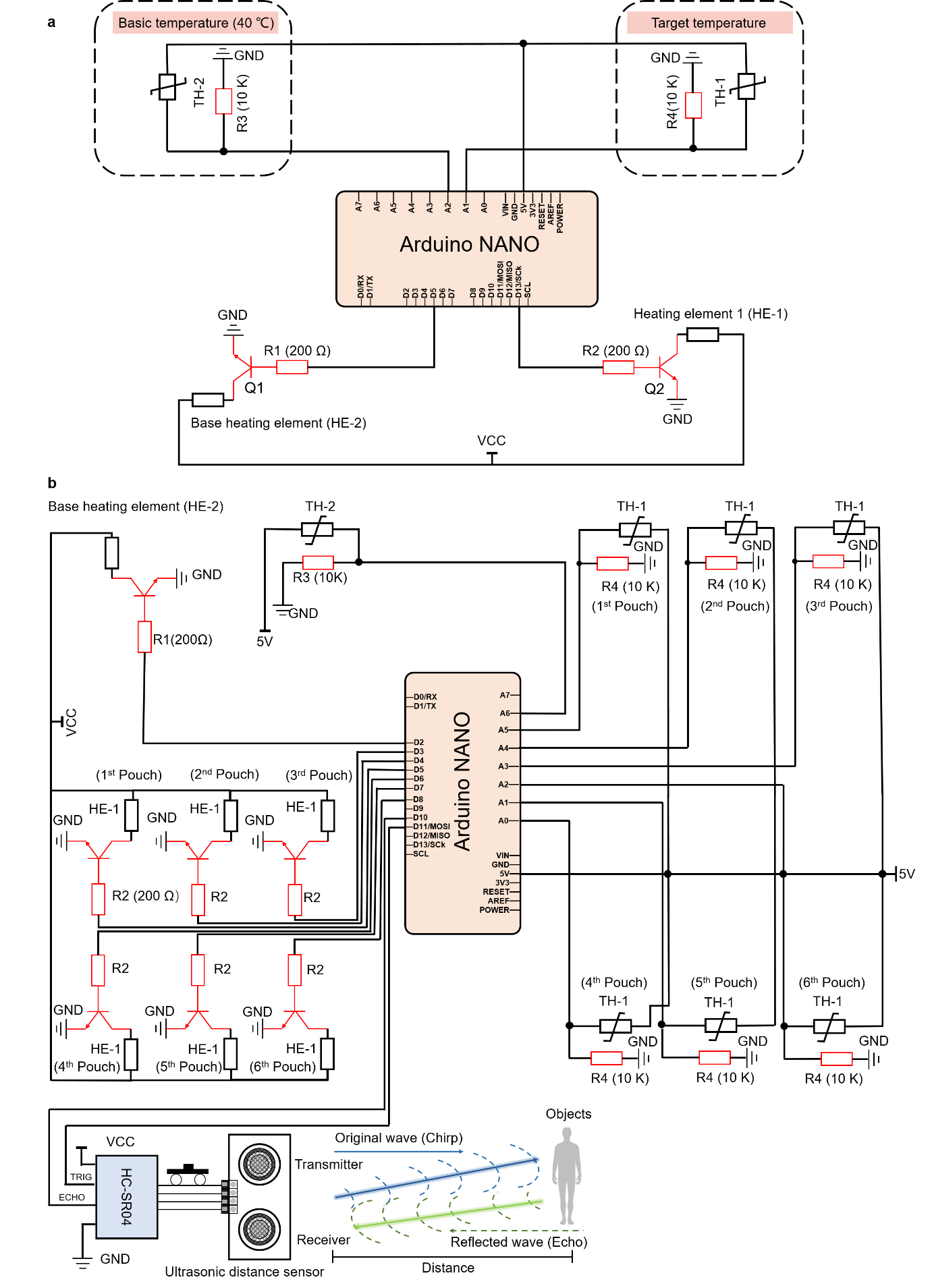


**Figure S7. Circuit diagrams for braille haptic devices. a,** The haptic units can be controlled by using a customized Arduino module-based circuit consisting of two heating elements (TH-1 and TH-2) and two thermistors (TH-1 and TH-2). **b**, The control circuit diagram of the 6-dot braille display. An ultrasonic distance sensor (HC-SR04, SparkFun Electronics) is used to detect the distance between the obstacle and the subject


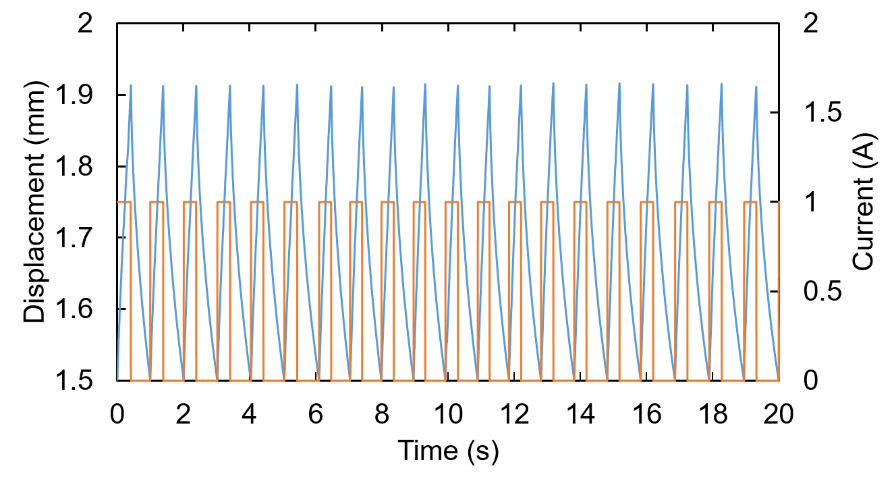


**Figure S8. Haptic unit with an actuating frequency of 1 Hz.** The stroke corresponding to the actuating frequency of 1Hz is around 0.43 mm.


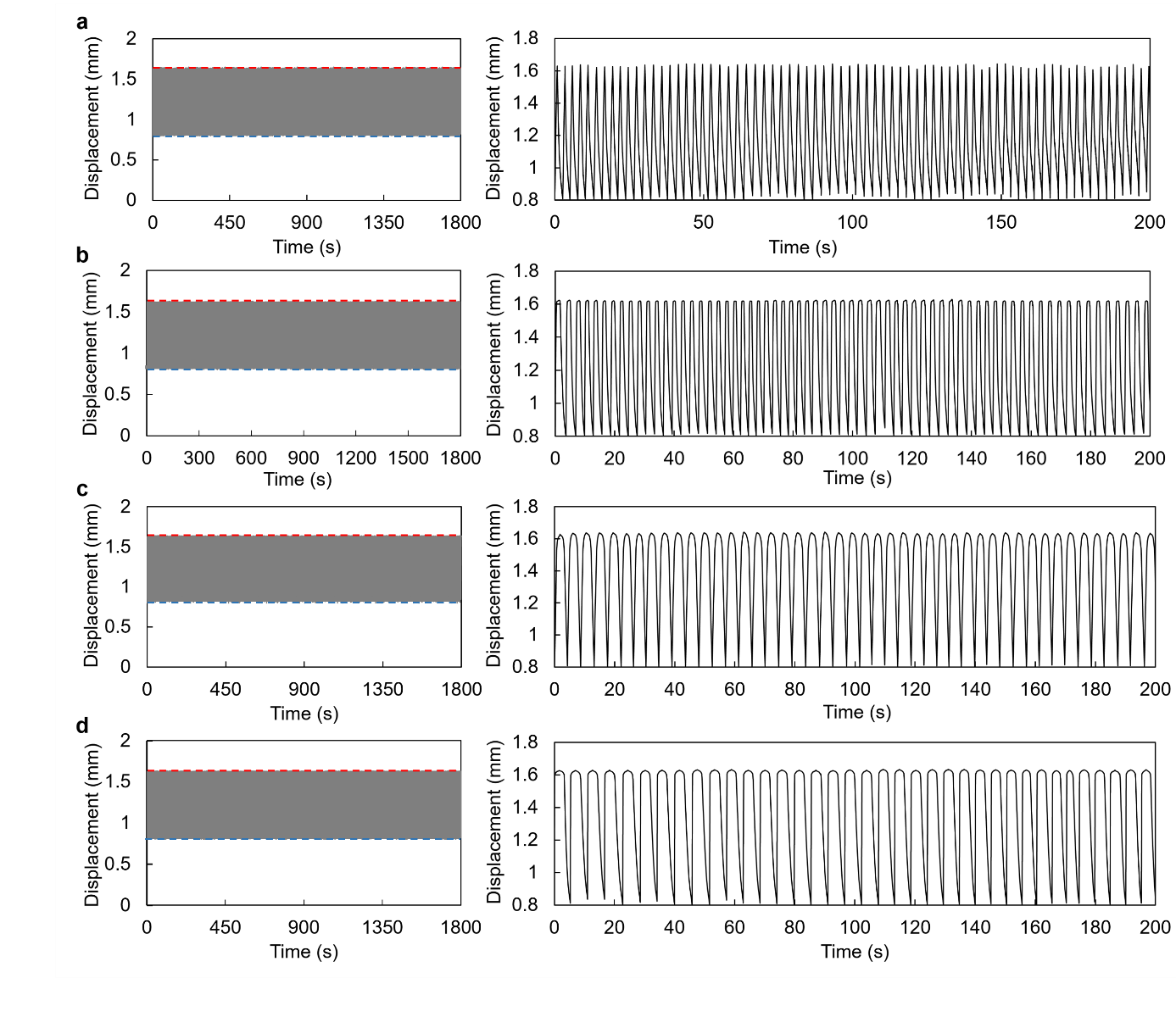


**Figure S9. Reciprocating motion of the haptic pin** within 1800 s (left) while maintaining peak position for 0 s (**a**), 1 s (**b**), 2 s (**c**), and 3 s (**d**) in each cycle, respectively. The graphs on the right show the details of the results by indicating the first 200 s of reciprocation of the haptic pin. The red dashed line represents the highest position of the haptic pin, and the blue dashed line represents the lowest position of the haptic pin.


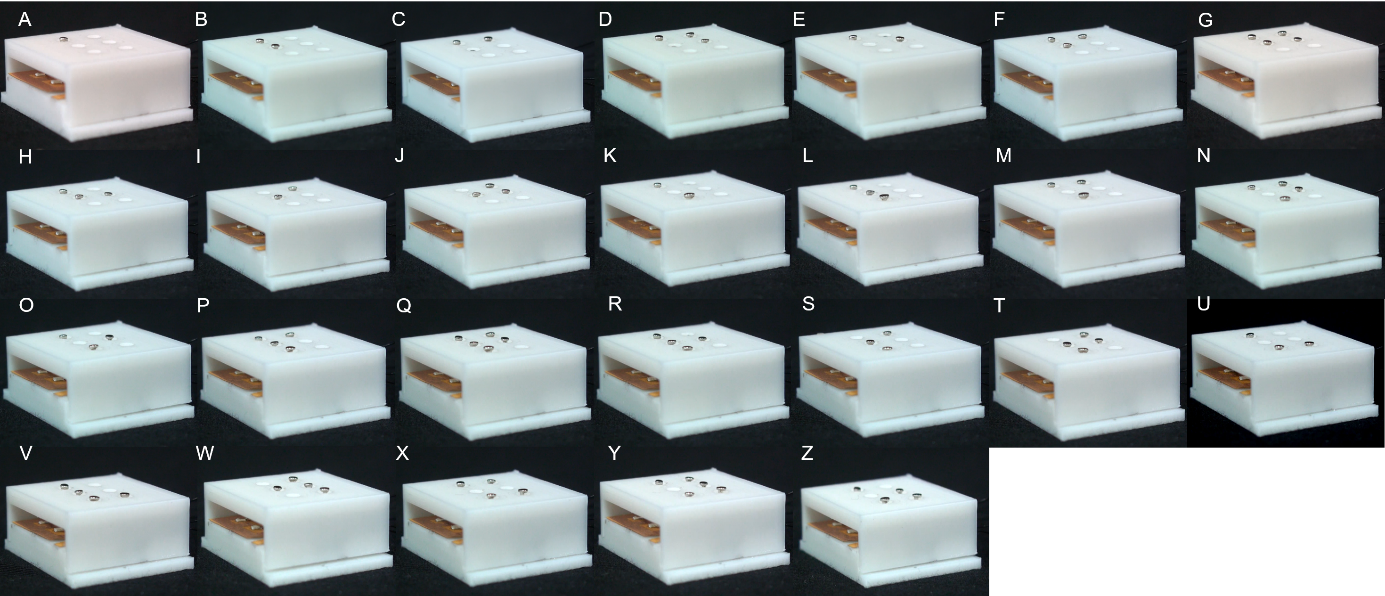


**Figure S10. Different display characters corresponding to Roman letters from A to Z.**


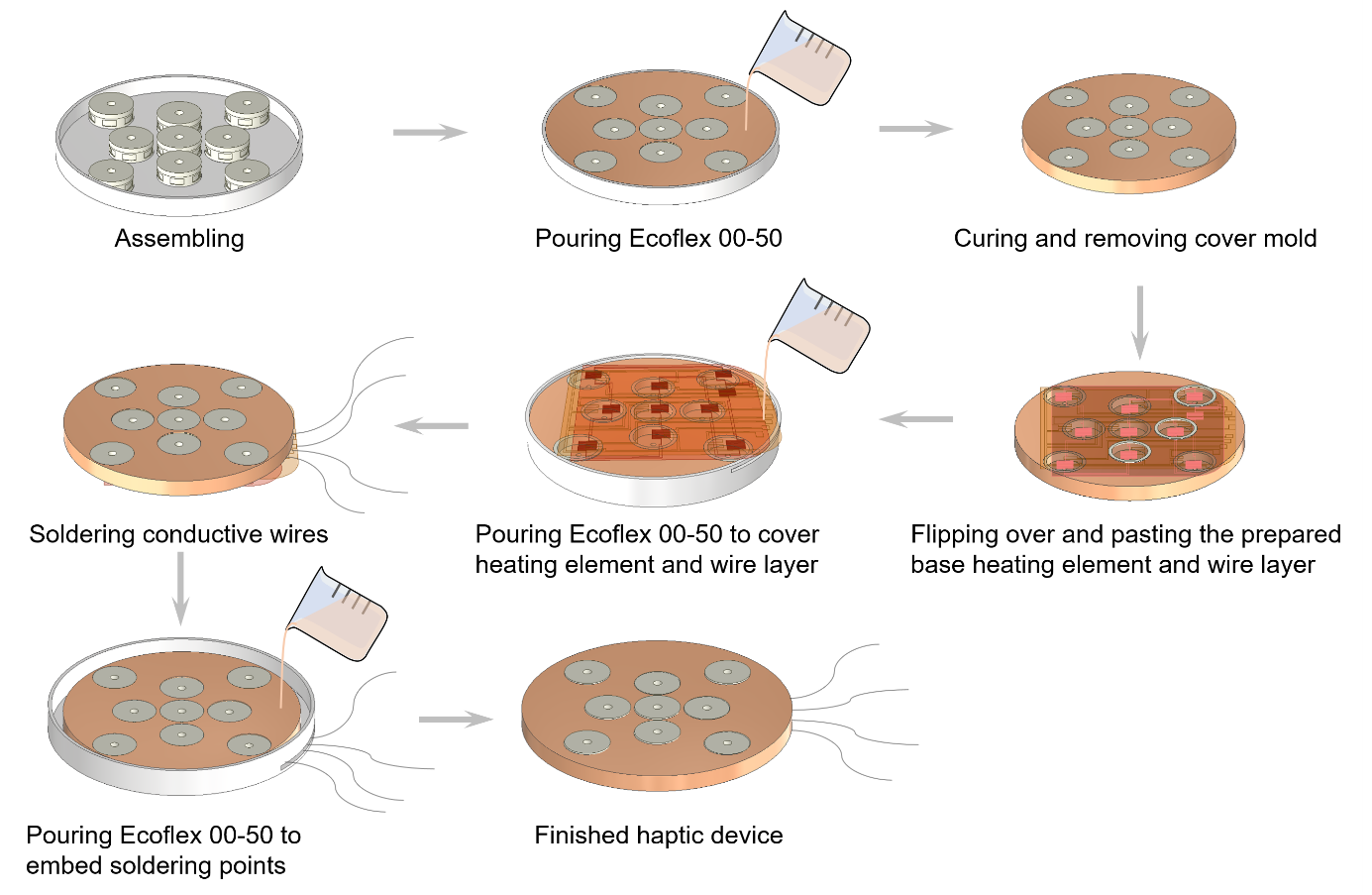


**Figure S11. Fabrication process of the flexible epidermal VR device.**

**
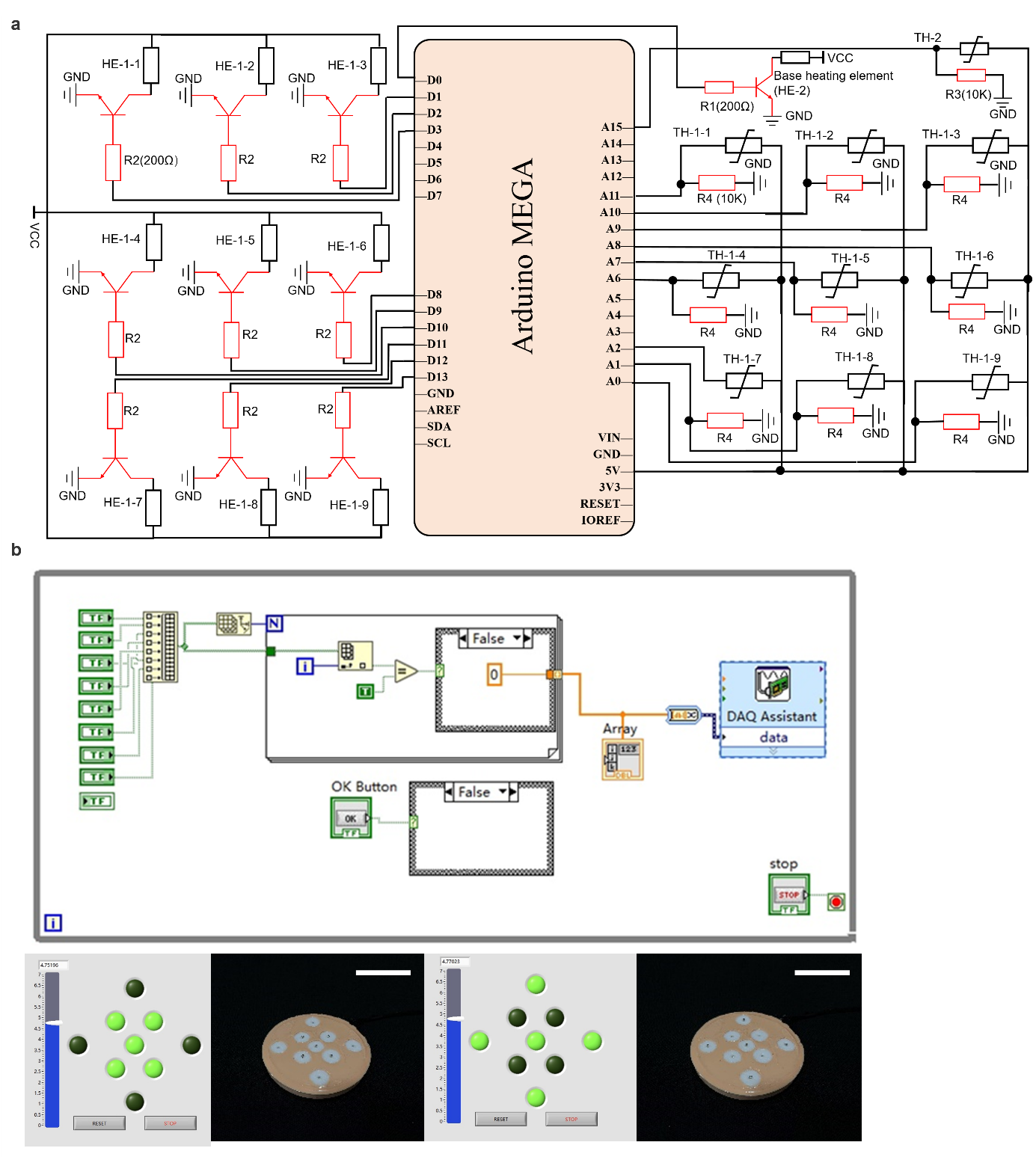
**

**Figure S12. Control diagrams for the epidermal VR device. a,** Circuit diagram for the 9-dot epidermal VR device. The heating elements and thermistors of the first to ninth pouch motor are named HE-1-1 to HE-1-9, and TH-1-1 to TH-1-9, respectively. **b,** Graphical interface of the control system based on the LabVIEW using NI-9263 (voltage output module) is designed for the flexible epidermal VR device. By using a graphical interface designed, the characteristics of the haptic stimulations can be controlled easily. As an example, two actuation patterns of the device are also described, where units in green are actuated and units in gray are in rest. All scale bars: 40 mm.

**Table S1. Main properties of the engineered fluid.**

| Parameters | Engineered fluid |
| --- | --- |
| The amount of fluid injected ($\mu$L)* | 1 |
| Liquid density (kg/m^3^) | 1510 |
| Vapor density (kg/m^3^) | 9.87 |
| Volume expansion factor * | 152.99 |
| Boiling points (℃) | 61 |
| Molecular weight (g/mol) | 250 |

* The approximate amount of the injected fluid is 1 $\mu$L, which is a much larger volume than that required for the maximum expansion of the pouch. The injected volume can not only compensate for fluid loss, but also can always ensure that a fully inflated state can be achieved.

**Movie S1. Haptic patterns of the braille handle.**

**Movie S2. Haptic patterns of the flexible epidermal VR device.**
